# Indocyanine green (ICG) angiography-guided thyroidectomy: description of surgical technique

**DOI:** 10.3389/fsurg.2023.1217764

**Published:** 2023-07-17

**Authors:** Pablo Moreno-Llorente, Mireia Pascua-Solé, Arantxa García-Barrasa, José Luis Muñoz-de-Nova

**Affiliations:** ^1^ Unit of Endocrine Surgery, Department of Surgery, Hospital Universitari de Bellvitge, Universitat de Barcelona (UB), Barcelona, Spain; ^2^ Department of General and Digestive Surgery, Hospital Universitario de la Princesa, Instituto de Investigación Sanitaria Princesa (IIS-IP), Universidad Autónoma de Madrid (UAM), Madrid, Spain

**Keywords:** indocyanine green angiography, angiography-guided thyroidectomy, parathyroid glands, hypoparathyroidism, vascular mapping

## Abstract

**Background:**

Postoperative hypoparathyroidism is the most common complication after total thyroidectomy and, when becomes permanent, lead to a myriad of clinical symptoms, long-term need of calcium and vitamin D supplementation and negative impact on the patient's health-related quality of life. Any surgical innovation that could reduce complications and improve outcomes of patients undergoing total thyroidectomy deserves to be considered. Angiography-Guided Thyroidectomy has been proposed as a modification of the standard technique of thyroidectomy aimed to identifying the vascular pattern of the parathyroid glands to maximize efforts for preserving functioning glands at the time of operation. Our aim is to provide a technical description of this procedure based on the use of indocyanine green (ICG) angiography to standardize this technique.

**Methods:**

The surgical steps that are followed during a total thyroidectomy are modified due to previous visualization of the feeding vessels of the parathyroid glands according to fluorescence of the vascular mapping obtained by ICG angiography prior to thyroidectomy. The first step is to perform an ICG angiography to assess anatomical features of the feeding vasculature of the parathyroid glands, which allows precise surgical dissection for preservation of the glands. Once the viability of the parathyroids has been evaluated angiographically, thyroidectomy is performed in a second step.

**Conclusions:**

ICG angiography-guided thyroidectomy may be effective to preserve the largest number of better perfused parathyroid glands, which would contribute to reduce the risk of postoperative and permanent hypoparathyroidism. It can be successfully and safely implemented in thyroid surgery and standardization of the technique is necessary to homogenize this procedure in the future, allowing a better comparation of the results to be published.

## 1. Introduction

Transient and/or permanent hypoparathyroidism is one of the most frequent complications after total thyroidectomy and results from direct injury, inadvertent resection or devascularization of the parathyroid glands (PGs) during surgery (1, 2). The rate of transient hypoparathyroidism is about 19%–38% (3). Permanent hypoparathyroidism (defined as the need of treatment beyond the first postoperative year) occurs in 0%–3% (4), although these figures may underestimate the incidence of hypoparathyroidism due to the lack of long-term follow-up data (3).

Intraoperative systematic search of the PGs during thyroidectomy has been associated with a higher incidence of episodes of hypocalcemia in the postoperative period, probably due to the damage of the vascular network that irrigates PGs when an extensive search is required (5). On the other hand, there is no doubt that the surgeon's experience is a determinant factor for the development of post-thyroidectomy transient or permanent episodes of hypocalcemia. However, it seems that both identification of the PGs and experience and skills of the surgeon for identifying and “*in situ*” parathyroid preservation are critical to prevent hypoparathyroidism, contributing to improve the quality of life of the patients (2).

Fluorescence with indocyanine green (ICG) has been used until now to assess the viability of the PGs, although ICG imaging for PG detection still lacks standardization (6–10). The initial studies evaluated the correlation of findings using intraoperative ICG with the postoperative function of the PGs, but results were inconclusive (11–14). Subsequent studies have shown that the diagnostic accuracy of hypocalcemia based on ICG fluorescence is similar to intraoperative measurements of intact parathyroid hormone (ioPTH) levels (15, 16) and that the use of ICG angiography during thyroidectomy increases preservation of the PGs (17, 18). In fact, the group of Benmiloud et al. (17) have the merit to suggest the potential benefits of this approach for the first time.

In conventional thyroidectomy, once the PGs have been identified, the difficulty lies in leaving them not only “*in situ*” but also well vascularized. In many cases, the feeding vessels of the glands cannot be visualized and surgical dissection is then performed intuitively. In order to avoid irreversible surgical maneuvers and in an attempt to preserve the glands and prevent hypoparathyroidism, we here propose the use of an ICG angiography for identifying the vascular supply of the PGs (vascular mapping) before thyroidectomy. The objective of the study is to describe the surgical technique of ICG angiography-guided thyroidectomy.

## 2. Surgical technique

### 2.1. Indications

Angiography with ICG can be used in patients undergoing thyroidectomy independently of indications for thyroid surgery. ICG arteriography-guided thyroidectomy is especially useful in the following conditions: (1) patients undergoing any thyroid surgery putting PGs at risk, particularly: total thyroidectomy for thyroid cancer in whom the risk of hypocalcemia is higher than in those treated with thyroidectomy for benign thyroid diseases especially when central neck dissection is performed and the inferior parathyroids may potentially become devascularized (19); (2) patients with large thyroid gland volume or retrosternal goiter in which only superior PGs could be identified; (3) patients undergoing reoperation for completion and/or central neck dissection in which ICG angiography-guided surgery can be useful for preserving vascularization of the PGs, although reoperations are associated with difficulties in identifying the PGs, for which autofluorescence could be a complementary aid (4). Furthermore, even in patients undergoing simple lobectomy, the use of ICG angiography for guiding this procedure will provide information on the viability of the PGs in the treated side in case of future neck surgery (completion thyroidectomy).

Exclusion criteria include patients with allergy to ICG components and contraindications for the use of ICG, such as hypersensitivity to the active principle or to sodium iodine, and patients with poor tolerability to a previous ICG injection.

### 2.2. Technical details

The ICG contrast material and a fluorescence imaging system are necessary. The contrast material is prepared by diluting a powdered vial of 25 mg ICG (Verdye®, Diagnostic Green GmbH, Aschheim-Dornach, Germany) in 10 ml of bidistilled water. The imaging system used in our center is a near-infrared camera (SPY-PHI®, Stryker Endoscopy, San Jose, CA, USA).

Total thyroidectomy is started with medialization of the thyroid lobe, with the minimal possible dissection to identify the PGs minimizing trauma to the glands or their vessels. In the upper pole, branches of the superior thyroid artery are sealed and cut on the anterior face of the thyroid capsule. Regarding the inferior pole, usually it is not necessary to ligate any vessel to expose the inferior PG or the thymic ligament. Finally, the inferior thyroid vein is ligated and cut over the carotid artery. Once the PGs in the first thyroid side have been identified, 1 ml of the contrast material is administered through a peripheral vein followed by 10 ml saline flush. After about 10–30 s, the contrast reaches the cervical region and the vascular pattern of the PGs and the vascular supply is enhanced, especially in those cases in which a defined pattern is identified. For the second side approached, the use of a 3 ml dose allows a proper identification of the vascular structures, independently of the previous dose.

**Figure 1:**
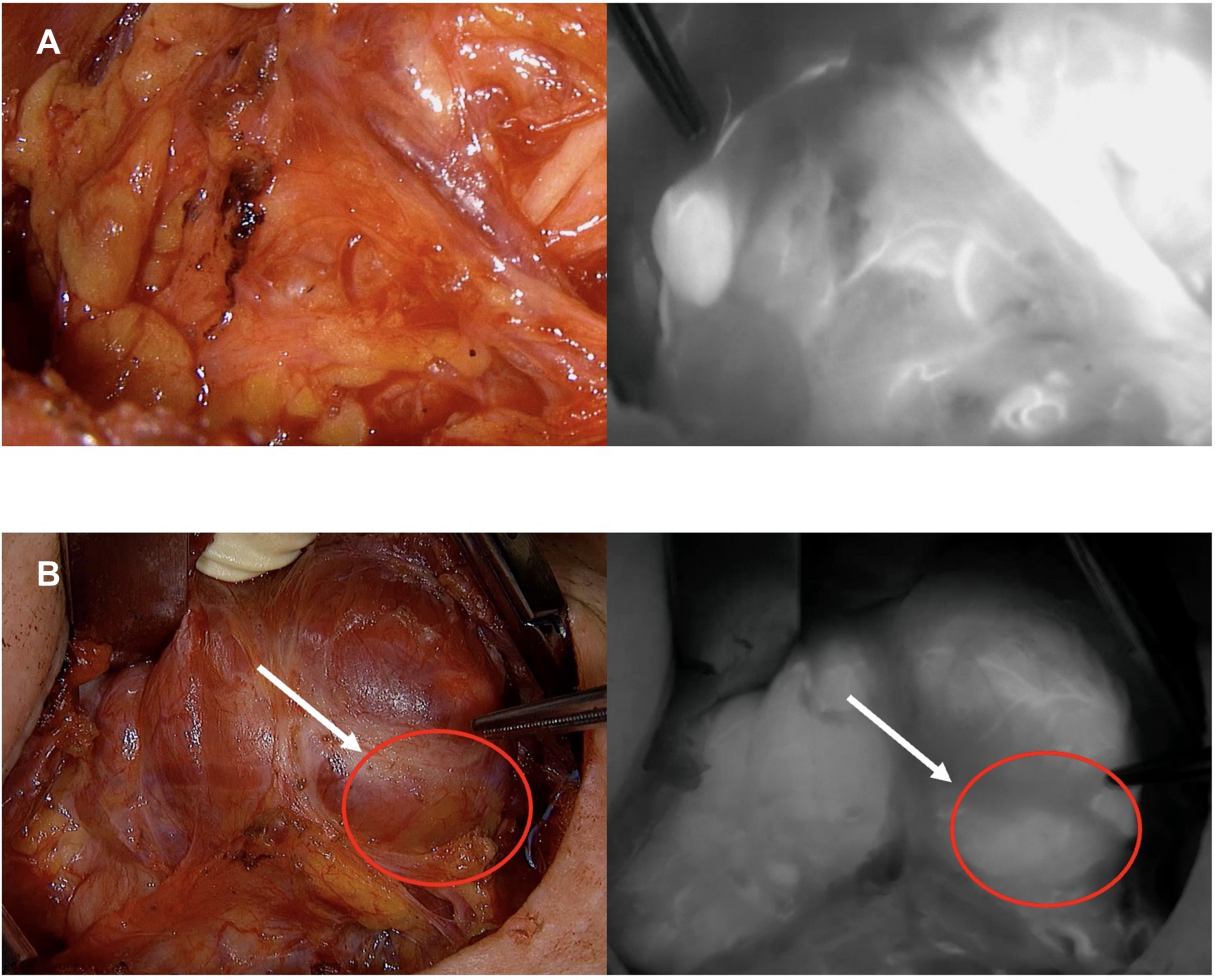
Types of vascular map pattern: (**A**) Defined; (**B**) Undefined.

The vascular pattern may include visualization of a clear feeding vessel with its pedicle or a diffuse vascular network around the PG without a clear feeding vessel (Figure 1). Once the vascular map has been identified, arteriography-guided thyroidectomy is performed. The initial objective is to preserve the parathyroid vessels until reaching the PG.

### 2.3. Surgical steps

#### 2.3.1. Right thyroidectomy

Once the vascular map of the PGs has been visualized, identification of the recurrent laryngeal nerve is an essential step and should be performed before dissecting the vessels. This is particularly crucial on the right side, where the branches of the inferior thyroid artery are commonly interspersed with the inferior laryngeal nerve. In case of difficulty, a safe alternative is to identify the recurrent laryngeal nerve in the lower third, dissecting it cranially until its intersection with the arterial vessels. In our practice, this is the initial step in patients undergoing central neck dissection or as a technical maneuver in cases in which the relation of the laryngeal nerve with the inferior thyroid artery or its branches cannot be easily identified. At this point, dissection is performed on the vessel that feeds the right inferior PG until reaching the gland. In those cases, in which the gland is located in the thyrothymic ligament, angiography may help to rule out whether the PG receives irrigation from the branches of the inferior thyroid artery in a caudal direction or from the mediastinum in a cranial direction.

**Figure 2:**
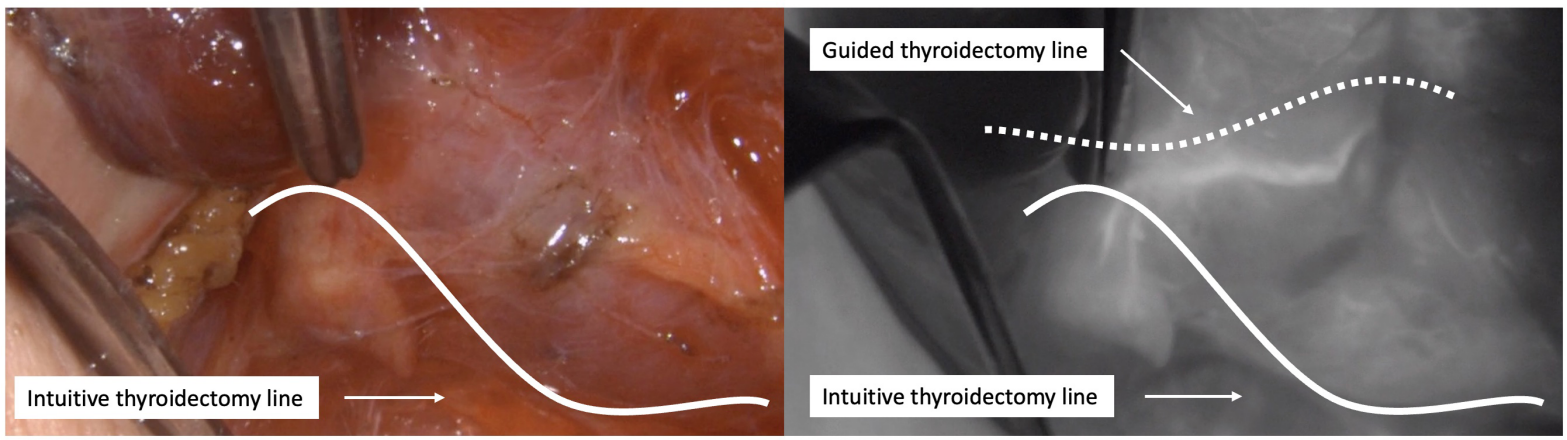
Dissection of the right superior PG. Left: intuitive thyroidectomy line based on the visual limits of the thyroid gland. Right: suggested thyroidectomy line based on ICG angiography.

Then, once the right inferior PG has been preserved and separated from the thyroid, dissection continues cranially from the crossing point of the inferior thyroid artery with the recurrent laryngeal nerve. In our experience, the superior glands are particularly susceptible to damage during thyroidectomy, especially when their feeding vessels come from the inferior thyroid artery, as they have a long course and are often intimately attached to the thyroid capsule, making their identification crucial (Figure 2).

Thereafter, once the right superior PG has been separated from the thyroid and the right recurrent laryngeal nerve has been left in a deep and medial plane, we proceed to complete the thyroidectomy while protecting the vessels and the nerve from the heat produced by the use of advanced energy devices. However, independently of the structures identified first (PGs or inferior laryngeal nerve), and in order to reduce the chance of injury of the vessels feeding PGs, the laryngeal nerve should not be dissected until parathyroid gland´s vessels have been identified. That is why when some PGs are identified late along the surgery, we need to inject a second dose of ICG to know the viability of their vessels.

**Figure 3:**
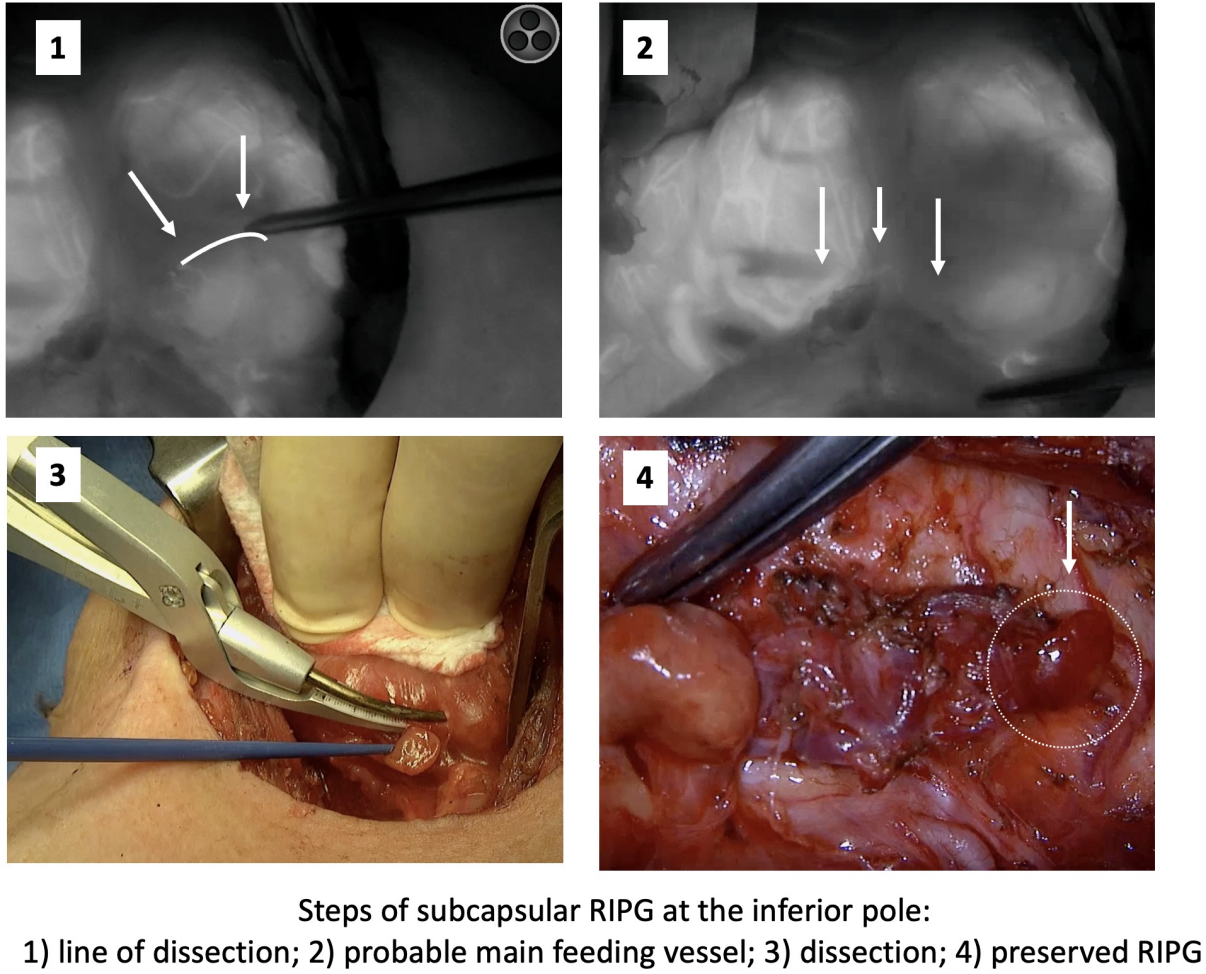
Subcapsular Right Inferior PG. Steps followed to preserve it based on angiography information.

Location of the right inferior PG in the subcapsular position of the lower pole of the thyroid lobe merits a special comment. In this position, there is a high probability of PGs becoming devascularized or being autotransplanted. In these cases, we propose first releasing the PG by sectioning the thyroid capsule and then proceeding to dissection of the feeding vessel until reaching its intersection with the recurrent laryngeal nerve (Figure 3).

In patients with large thyroid volumes, identification of both the PGs and the feeding vessels are greatly limited by the volume of the thyroid gland and the anatomical location of the PGs. Because the superior PGs have a more constant anatomical location, it is advisable starting the dissection at the superior pole given that identification of the inferior PGs will be more difficult.

#### 2.3.2. Left thyroidectomy

On the left side, the left recurrent laryngeal nerve in the tracheoesophageal groove is first identified and then the inferior thyroid artery. At this point, vessels are dissected up to the inferior and/or superior PGs.

#### 2.3.3. Evaluation of PG viability

**Figure 4:**
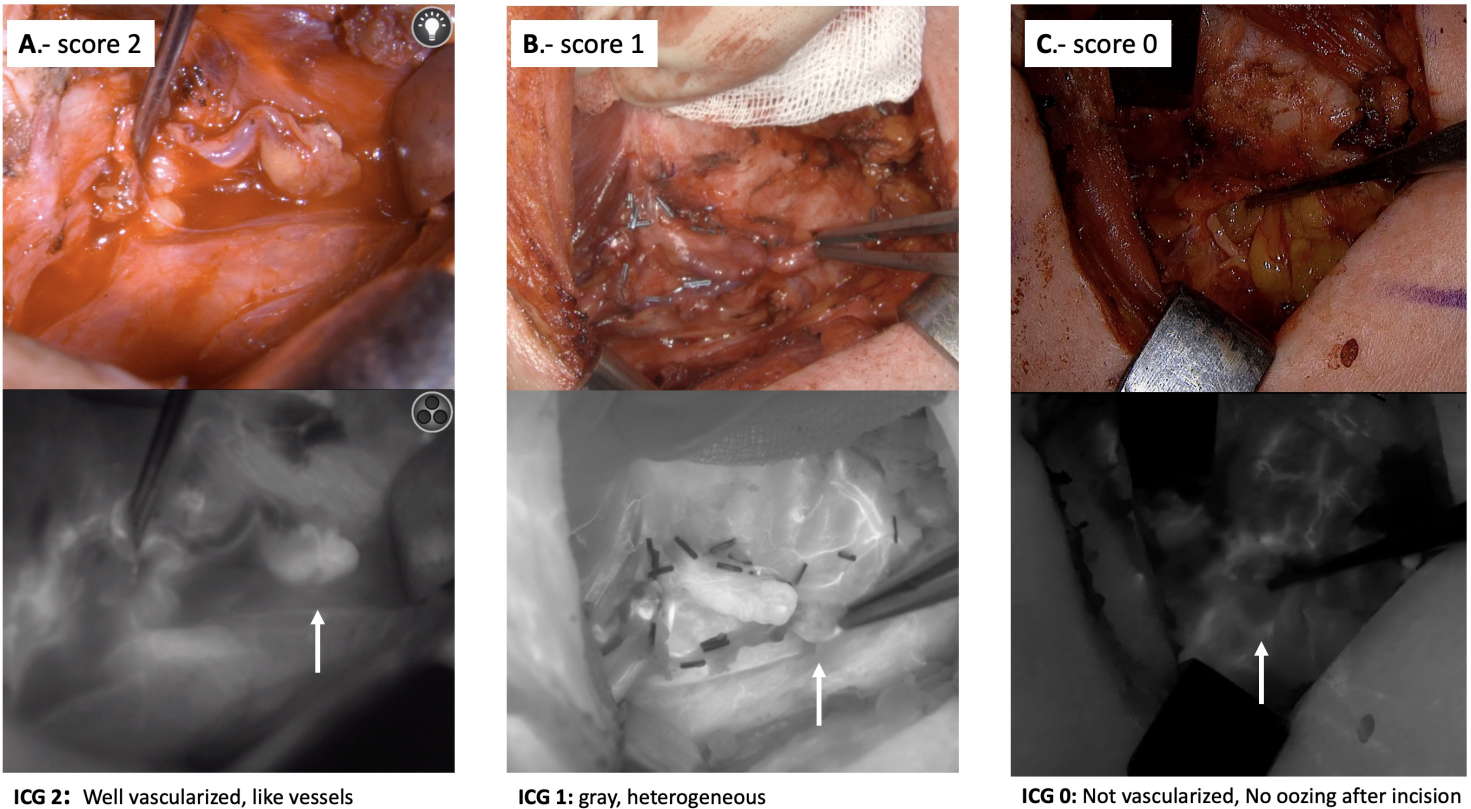
Intraoperative ICG angiographic images, showing examples of parathyroid glands well vascularized (white, viability score 2), partially vascularized (gray/heterogeneous, viability score 1) and devascularized (black, viability score 0).

After lobectomy, 3 ml of ICG are administered to evaluate the viability of the PGs using a black and white scale (0, 1, and 2). The color of the gland reflecting the degree of perfusion can vary from black (suggesting that it is not vascularized and likely non-viable) to white (suggesting that it is well vascularized and viable). Accordingly, glands are categorized as 0, black (nonvascularized); 1, gray/heterogeneous (partially vascularized); and 2, white (well vascularized), see Figure 4.

The existence of a residual amount of ICG after the first dose is not a drawback for further enhancement of the PGs and their feeding vessels using subsequent 3 ml ICG doses administered at least at 5 min intervals. Doses can be repeated up to a maximum of 5 mg/kg. These doses, however, are far from the maximum allowed doses, since for a patient of 70 kg taking into account that 2.5 mg/ml are obtained when preparing a vial, the maximum dose to be administered would be 350 mg and equivalent to 140 ml.

When central neck dissection is performed, this protocol should be performed at the end of the procedure on each side.

In our experience, a single PG with an ICG score 2 is sufficient for preventing postoperative hypocalcemia (15). In daily practice, although we still correlate ICG scoring with ioPTH levels (using a chemiluminescent immunometric assay) taken 10 min after completion of total thyroidectomy and 10 min after central neck dissection, the criterion of an ICG score 2 prevails over a significant decrease of ioPTH for routine management in the immediate postoperative period, assuming that the patient will not develop hypocalcemia.

## 3. Discussion

The use of ICG angiography to identify vascular mapping of the PGs during thyroid surgery is a recently described surgical procedure (17). Here we describe the angiography-guided thyroidectomy as a modification of the conventional surgical procedure that proposes a conceptual change, i.e., prioritizing first the identification and preservation of the GPs and their nutritional vessels and then completing the thyroidectomy. In conventional thyroidectomy, once the PGs are identified, the surgeon intuitively establishes an imaginary line of thyroidectomy attempting to leave the PGs “*in situ*” without managing to identify in most cases the vessels feeding the PGs, and without evidencing the functioning of the glands beyond their macroscopic appearance. However, in a previous study of our group, visual macroscopic assessment of the PGs even performed by senior surgeons had a lower specificity and predictive values as compared with ioPTH and ICG score 2 in predicting early post-thyroidectomy hypocalcemia (15).

Knowing the vascular mapping of the PGs before performing thyroidectomy allows the surgical procedure to be started by prioritizing preservation of the feeding parathyroid vessels, then isolating the glands from the thyroid or the recurrent laryngeal nerve and completing the thyroidectomy in a final step. Regarding the ICG doses, the dose of 1 ml of ICG was arbitrarily selected as in our experience it was the minimum amount of ICG offering clear visualization of the PGS and its feeding vessels. All the remaining doses (3 ml each) were taken from the fists studies from Vidal et al. (7, 16) that were also used in all our previous published papers (15, 18). However, final consensus regarding the ICG doses has not been published yet.

Postoperative permanent hypoparathyroidism is a chronic disease with a negative impact on the quality of life (20, 21), difficulties for patients in compliance to lifelong calcium and vitamin D replacement therapy (22), recurrent visits to the emergency department for acute symptomatic hypocalcemia and comorbidities (23, 24), and substantial financial burden due to increased health care resource utilization (25).

Actively searching the PGs during thyroid surgery may cause inadvertent damage of the glands or their blood supply and therefore leading to hypoparathyroidism (26, 27). ICG angiography-guided thyroidectomy is a tool that helps the surgeon in preserving well-vascularized PGs. Our experience is consistent with data reported in the study of Benmiloud et al. (17) in which ICG-based intraoperative mapping angiograms of the PGs allowed for the preservation of more well-perfused glands. A recently published study by our group, conducted on a series of 120 patients undergoing total thyroidectomy, has demonstrated a statistically significant decrease in both immediate and permanent hypoparathyroidism among patients included in the ICG angiography-guided thyroidectomy group as compared with a historical cohort group (18).

In conclusion, this report aims to introduce ICG angiography-guided thyroidectomy, an innovative surgical technique that has allowed decreasing the rates of hypoparathyroidism in our center. These promising preliminary results should be confirmed in a randomized single-blind controlled multicenter trial, the results of which will strongly support to adopt this new surgical strategy in the field of thyroid surgery (28).

## Data Availability

The original contributions presented in the study are included in the article/Supplementary Material, further inquiries can be directed to the corresponding author.
